# Supplementary material for: The EnteroBase user's guide, with case studies on Salmonella transmissions, Yersinia pestis phylogeny, and Escherichia core genomic diversity
Source: Genome Res. 2020 Jan;30(1):138–52. doi: 10.1101/gr.251678.119 (PMC6961584; doi:10.1101/gr.251678.119)
Supplement: Supplemental Material [file supp_30_1_138__index.html]

The EnteroBase user's guide, with case studies on Salmonella transmissions, Yersinia pestis phylogeny, and Escherichia core genomic diversity — Supplemental Material 

# The EnteroBase user's guide, with case studies on *Salmonella* transmissions, *Yersinia pestis* phylogeny, and *Escherichia* core genomic diversity

## Supplemental Material

- Supplemental\_Code.zip
- Supplemental\_Material.pdf
