# Supplementary material for: The EnteroBase user's guide, with case studies on Salmonella transmissions, Yersinia pestis phylogeny, and Escherichia core genomic diversity
Source: Genome Res. 2020 Jan;30(1):138–52. doi: 10.1101/gr.251678.119 (PMC6961584; doi:10.1101/gr.251678.119)
Supplement: Supplemental Material [file supp_gr.251678.119_Supplemental_Material.pdf]

Supplemental Material

EnteroBase Metaparser conversions of metadata for host or environment.

Metadata within short read archives are associated with a wide range of designations for the hosts or environments from which the bacteria were isolated. This raw data was stored in EnteroBase as Source->Details. In 2015, we manually assigned 3,546 distinct Source->Details entries to pre-defined categories of Source->Niche and Source->Type (Supplemental Fig. S3; Supplemental Table S4). 2,000 entries provided an initial training set and the remaining 1,546 entries served as a test set for a Native Bayesian (NB) source classifier that is implemented in the NLTK Natural Language Toolkit for Python (Bird *et al.* 2009). Two classifiers were trained and evaluated independently for Source->Niche and Source->Type. The NB source classifier achieved an accuracy of ~80% on the test set after initial training. We then re-trained the NB source classifier using all 3,546 manually curated entries, and used it continuously until October 2019 to assign GenBank metadata into the nested Source metadata fields within EnteroBase.

Over that time period, manual curation revealed a number of obvious misclassifications, which we corrected manually. However the number of existing EnteroBase curators is insufficient to support the manual curation of the 100,000s of entries in EnteroBase. We retested the NB source classifier in 2018 with an independent, recent set of 3,000 manually curated entries. Those tests yielded an accuracy of only 60%, which reflects the large number of novel designations which are currently being used. Many terms were not recognized by the source classifier because they were not included in the initial training set. We therefore developed and implemented a new FT Metaparser in Oct. 2019. It is based on the Python FastText classifier library (Joulin *et al.* 2017), with a pre-loaded word vector that was trained on Wikipedia in 2016 (Bojanowski *et al.* 2017). After training it as above on 3,577 curated metadata sets, the FT Metaparser achieved an accuracy of ~93% on a test set of 894 independent metadata records. We anticipate better long-term sustainability for the FT parser, and are currently evaluating it with additional data from independent, external sources. The FT Metaparser will be released as part of a separate publication together with the detailed evaluation procedures.

An example of using Uberstrains and sub-strains.

The EnteroBase *Yersinia* database includes two distinct genomes for *Y. pestis* CO92, the genome originally sequenced in 2001 (Parkhill et al. 2001) and a subsequent, corrected genome from 2015 (Johnson et al. 2015). The more recent genome sequence is the Uberstrain for genomes from multiple bacterial colonies that were sequenced after infecting prairie dogs in the laboratory with CO92 (project PRJNA340278) (Supplemental Fig. S4). The older genome is maintained in EnteroBase as a separate Uberstrain because it was previously used as the reference genome for SNP calling (Morelli et al. 2010; Bos et al. 2011), and continues to be used as the reference for *Y. pestis* ancient DNA (aDNA) projects based on archaeological samples (Wagner et al. 2014; Rasmussen et al. 2015; Bos et al. 2016; Feldman et al. 2016; Spyrou et al. 2016; Spyrou et al. 2018; Margaryan et al. 2018). Fig. S4 illustrates these points, as well as showing how to load and display sub-strains and Uberstrains,

Micro-epidemiology of Agama transmissions between humans and countries.

The additional Agama genomes provided by the Agama Study Group included isolates from humans in Ireland, France or Austria, as well as multiple isolates from animals and food. The additional genomes showed that HC100_299 was also isolated from humans in Ireland as well as from dogs, cows and horses (Fig. 4B). All isolates in HC100_67355 were from Ireland, consisting of one clade of 12 isolates from humans and a second clade containing one isolate from mussels. HC100_2433 now contained not only isolates from the British Isles, but also isolates from France and Austria. We were particularly struck by four genomes in HC5_140035 (Fig. 4B, green arrow at 04:00), which had all been isolated in 2018. Three of these isolates were from Austria, two from frozen chives and one from a blood culture from a case of human septicemia. The Austrian isolates differ from each other in pair-wise comparisons by 2-5 non-repetitive core SNPs and 2-4 cgMLST loci. The fourth isolate in HC5_140035 was from a human in France. It differed by 5 SNPs and 5 cgMLST loci from the three Austrian isolates and by 8-35 SNPs and 6-23 cgMLST loci from other Agama in France. We do not know of any epidemiological data that support food-borne transmission of these organisms between France and Austria, or

that indicate that frozen chives are a vehicle for food-borne invasive Agama disease. However, this observation is a strong signal for recent transmission chains of Agama between France and Austria with the possible involvement of frozen food products.

Comparison of Clermont typing with HC1100 clustering

EnteroBase provides phylotype predictions for *Escherichia* based on Clermont typing according to two distinct algorithms (Beghain *et al.* 2018; Waters *et al.* 2018). The assignments by both Clermont typing algorithms are largely congruent. And those assignments are also largely congruent with the general structure of the ML tree (Fig. S9 inset). However, Clermont typing is based on the presence/absence of accessory genes, which are more variable than are sequences of the core genome. As a result, close examination of the ML tree shows multiple exceptions, as indicated by flashes of discrepant colors in Fig. S8. Clermont typing never included *S. flexneri* (HC1100_192) and scores it incorrectly as haplogroup A. Clermont typing also never included *S. sonnei,* which it scores as Unknown (Fig. S8). Until recently, Clermont typing also scored haplogroup G as haplogroups F (Clermont *et al.* 2019), but this has now been corrected in the most recent version of the ClermonTyping software,

Supplementary Reference List

Achtman M, Mercer A, Kusecek B, Pohl A, Heuzenroeder M, Aaronson W, Sutton A, Silver RP. 1983. Six widespread bacterial clones among *Escherichia coli* K1 isolates. *Infect Immun* **39**: 315-335.

Achtman M and Zhou Z. 2019. Analysis of the human oral microbiome from modern and historical samples with SPARSE and EToKi. *BioRxiv* 842542.

Altschul SF, Gish W, Miller W, Myers EW, Lipman DJ. 1990. Basic local alignment search tool. *J Mol Biol* **215**: 403-410.

Argimon S, Abudahab K, Goater RJ, Fedosejev A, Bhai J, Glasner C, Feil EJ, Holden MT, Yeats CA, Grundmann H, et al. 2016. Microreact: visualizing and sharing data for genomic epidemiology and phylogeography. *Microb Genom* **2**: e000093.

Bankevich A, Nurk S, Antipov D, Gurevich AA, Dvorkin M, Kulikov AS, Lesin VM, Nikolenko SI, Pham S, Prjibelski AD, et al. 2012. SPAdes: a new genome assembly algorithm and its applications to single-cell sequencing. *J Comput Biol* **19**: 455-477.

Beghain J, Bridier-Nahmias A, Le NH, Denamur E, Clermont O. 2018. ClermonTyping: an easy-to-use and accurate *in silico* method for *Escherichia* genus strain phylotyping. *Microb Genom* **4**.

Ben Zakour NL, Alsheikh-Hussain AS, Ashcroft MM, Khanh Nhu NT, Roberts LW, Stanton-Cook M, Schembri MA, Beatson SA. 2016. Sequential acquisition of virulence and fluoroquinolone resistance has shaped the evolution of *Escherichia coli* ST131. *MBio* **7**: e00347-16.

Benson G. 1999. Tandem repeats finder: a program to analyze DNA sequences. *Nucleic Acids Res* **27**: 573-580.

Bird S, Klein E, Loper E. 2009. *Natural Language Processing with Python: Analyzing Text with the Natural Language Toolkit*, 1 edition. O'Reilly Media, Sebastopol, CA.

Bojanowski P, Grave E, Joulin A, Mikolov T. 2017. Enriching word vectors with subword information. *Transactions of the Association for Computational Linguistics* **5**: 135-146.

Bos KI, Herbig A, Sahl J, Waglechner N, Fourment M, Forrest SA, Klunk J, Schuenemann VJ, Poinar D, Kuch M, et al. 2016. Eighteenth century *Yersinia pestis* genomes reveal the long-term persistence of an historical plague focus. *Elife* **5**.

Bos KI, Schuenemann VJ, Golding GB, Burbano HA, Waglechner N, Coombes BK, McPhee JB, Dewitte SN, Meyer M, Schmedes S, et al. 2011. A draft genome of *Yersinia pestis* from victims of the Black Death. *Nature* **478**: 506-510.

Bushnell B. BBMap short read aligner. 2016. *URL*

Clermont O, Dixit OVA, Vangchhia B, Condamine B, Dion S, Bridier-Nahmias A, Denamur E, Gordon D. 2019. Characterization and rapid identification of phylogroup G in *Escherichia coli,* a lineage with high virulence and antibiotic resistance potential. *Environ Microbiol* **21**: 3107-3117.

Dallman TJ, Ashton PM, Byrne L, Perry NT, Petrovska L, Ellis R, Allison L, Hanson M, Holmes A, Gunn GJ, et al. 2015. Applying phylogenomics to understand the emergence of Shiga-toxin-

producing *Escherichia coli* O157:H7 strains causing severe human disease in the UK. *Microb Genom* **1**: e000029.

DebRoy C, Fratamico PM, Yan X, Baranzoni G, Liu Y, Needleman DS, Tebbs R, O'Connell CD, Allred A, Swimley M, et al. 2016. Correction: Comparison of O-Antigen Gene Clusters of All O-Serogroups of Escherichia coli and Proposal for Adopting a New Nomenclature for O-Typing. *PLoS ONE* **11**: e0154551.

Edgar RC. 2007. PILER-CR: fast and accurate identification of CRISPR repeats. *BMC Bioinformatics* **8**: 18.

Edgar RC. 2010. Search and clustering orders of magnitude faster than BLAST. *Bioinformatics* **26**: 2460-2461.

Feldman M, Harbeck M, Keller M, Spyrou MA, Rott A, Trautmann B, Scholz HC, Paffgen B, Peters J, McCormick M, et al. 2016. A high-coverage *Yersinia pestis* genome from a sixth-century Justinianic plague victim. *Mol Biol Evol* **33**: 2911-2923.

Gordon DM, Geyik S, Clermont O, O'Brien CL, Huang S, Abayasekara C, Rajesh A, Kennedy K, Collignon P, Pavli P, et al. 2017. Fine-scale structure analysis shows epidemic patterns of Clonal Complex 95, a cosmopolitan *Escherichia coli* lineage responsible for extraintestinal infection. *mSphere* **2**.

Joensen KG, Tetzschner AM, Iguchi A, Aarestrup FM, Scheutz F. 2015. Rapid and easy *in silico* serotyping of *Escherichia coli* isolates by use of whole-genome sequencing data. *J Clin Microbiol* **53**: 2410-2426.

Johnson JR, Davis G, Clabots C, Johnston BD, Porter S, DebRoy C, Pomputius W, Ender PT, Cooperstock M, Slater BS, et al. 2016. Household clustering of *Escherichia coli* sequence type 131 clinical and fecal isolates according to whole genome sequence analysis. *Open Forum Infect Dis* **3**: ofw129.

Johnson SL, Daligault HE, Davenport KW, Jaissle J, Frey KG, Ladner JT, Broomall SM, Bishop-Lilly KA, Bruce DC, Coyne SR, et al. 2015. Thirty-two complete genome assemblies of nine *Yersinia* species, Including *Y. pestis*, *Y. pseudotuberculosis*, and *Y. enterocolitica*. *Genome Announc* **3**.

Johnson TJ, Elnekave E, Miller EA, Munoz-Aguayo J, Flores FC, Johnston B, Nielson DW, Logue CM, Johnson JR. 2019. Phylogenomic analysis of extraintestinal pathogenic *Escherichia coli* Sequence Type 1193, an emerging multidrug-resistant clonal group. *Antimicrob Agents Chemother* **63**.

Joulin A, Grave E, Bojanowski P, Mikolov T. Bag of tricks for efficient text classification. 2017. *Proceedings of the 15th Conference of the {E}uropean Chapter of the Association for Computational Linguistics* **2:** 427-431. Valencia, Spain, Association for Computational Linguistics.

Katoh K and Standley DM. 2013. MAFFT multiple sequence alignment software version 7: improvements in performance and usability. *Mol Biol Evol* **30**: 772-780.

Kielbasa SM, Wan R, Sato K, Horton P, Frith MC. 2011. Adaptive seeds tame genomic sequence comparison. *Genome Res* **21**: 487-493.

Leopold SR, Magrini V, Holt NJ, Shaikh N, Mardis ER, Cagno J, Ogura Y, Iguchi A, Hayashi T, Mellmann A, et al. 2009. A precise reconstruction of the emergence and constrained radiations of *Escherichia coli* O157 portrayed by backbone concatenomic analysis. *Proc Natl Acad Sci USA* **106**: 8713-8718.

Li H and Durbin R. 2010. Fast and accurate long-read alignment with Burrows-Wheeler transform. *Bioinformatics* **26**: 589-595.

Liu CM, Stegger M, Aziz M, Johnson TJ, Waits K, Nordstrom L, Gauld L, Weaver B, Rolland D, Statham S, et al. 2018. *Escherichia coli* ST131-H22 as a foodborne uropathogen. *MBio* **9**.

Luo C, Walk ST, Gordon DM, Feldgarden M, Tiedje JM, Konstantinidis KT. 2011. Genome sequencing of environmental *Escherichia coli* expands understanding of the ecology and speciation of the model bacterial species. *Proc Natl Acad Sci USA* **108**: 7200-7205.

Margaryan A, Hansen HB, Rasmussen S, Sikora M, Moiseyev V, Khoklov A, Epimakhov A, Yepiskoposyan L, Kriiska A, Varul L, et al. 2018. Ancient pathogen DNA in human teeth and petrous bones. *Ecol Evol* **8**: 3534-3542.

McDonald JL, Robertson A, Silk MJ. 2018. Wildlife disease ecology from the individual to the population: Insights from a long-term study of a naturally infected European badger population. *J Anim Ecol* **87**: 101-112.

Morelli G, Song Y, Mazzoni CJ, Eppinger M, Roumagnac P, Wagner DM, Feldkamp M, Kusecek B, Vogler AJ, Li Y, et al. 2010. *Yersinia pestis* genome sequencing identifies patterns of global phylogenetic diversity. *Nature Genet* **42**: 1140-1143.

Parkhill J, Wren BW, Thomson NR, Titball RW, Holden MT, Prentice MB, Sebaihia M, James KD, Churcher C, Mungall KL, et al. 2001. Genome sequence of *Yersinia pestis*, the causative agent of plague. *Nature* **413**: 523-527.

Price MN, Dehal PS, Arkin AP. 2010. FastTree 2--approximately maximum-likelihood trees for large alignments. *PLoS One* **5**: e9490.

Rasmussen S, Allentoft ME, Nielsen K, Orlando L, Sikora M, Sjogren KG, Pedersen AG, Schubert M, Van DA, Kapel CM, et al. 2015. Early divergent strains of *Yersinia pestis* in Eurasia 5,000 years ago. *Cell* **163**: 571-582.

Spyrou MA, Tukhbatova RI, Feldman M, Drath J, Kacki S, Beltran de HJ, Arnold S, Sitdikov AG, Castex D, Wahl J, et al. 2016. Historical *Y. pestis* genomes reveal the European black death as the source of ancient and modern plague pandemics. *Cell Host Microbe* **19**: 874-881.

Spyrou MA, Tukhbatova RI, Wang CC, Valtuena AA, Lankapalli AK, Kondrashin VV, Tsybin VA, Khokhlov A, Kuhnert D, Herbig A, et al. 2018. Analysis of 3800-year-old *Yersinia pestis* genomes suggests Bronze Age origin for bubonic plague. *Nat Commun* **9**: 2234.

Stamatakis A. 2014. RAxML version 8: a tool for phylogenetic analysis and post-analysis of large phylogenies. *Bioinformatics* **30**: 1312-1313.

Stoesser N, Sheppard AE, Pankhurst L, De MN, Moore CE, Sebra R, Turner P, Anson LW, Kasarskis A, Batty EM, et al. 2016. Evolutionary history of the global emergence of the *Escherichia coli* epidemic clone ST131. *MBio* **7**: e02162.

van der Putten BCL, Matamoros S, COMBAT consortium, Schultsz C. 2019. Genomic evidence for revising the *Escherichia* genus and description of *Escherichia ruysiae sp. nov. BioRxiv* 781724.

Wagner DM, Klunk J, Harbeck M, Devault A, Waglechner N, Sahl JW, Enk J, Birdsell DN, Kuch M, Lumibao C, et al. 2014. *Yersinia pestis* and the plague of Justinian 541-543 AD: a genomic analysis. *Lancet Infect Dis* **14**: 319-326.

Walker BJ, Abeel T, Shea T, Priest M, Abouelleil A, Sakthikumar S, Cuomo CA, Zeng Q, Wortman J, Young SK, et al. 2014. Pilon: an integrated tool for comprehensive microbial variant detection and genome assembly improvement. *PLoS ONE* **9**: e112963.

Waters NR, Abram F, Brennan F, Holmes A, Pritchard L. 2018. Easily phylotyping *E. coli* via the EzClermont web app and command-line tool. *BioRxiv* 317610.

Wirth T, Falush D, Lan R, Colles F, Mensa P, Wieler LH, Karch H, Reeves PR, Maiden MC, Ochman H, et al. 2006. Sex and virulence in *Escherichia coli*: an evolutionary perspective. *Mol Microbiol* **60**: 1136-1151.

Wood DE and Salzberg SL. 2014. Kraken: ultrafast metagenomic sequence classification using exact alignments. *Genome Biol* **15**: R46.

Zhou Z, Luhmann N, Alikhan N-F, Quince C, Achtman M. 2018. Accurate reconstruction of microbial strains from metagenomic sequencing using representative reference genomes. In RECOMB 2018 , pp. 225-240. Springer, Cham.

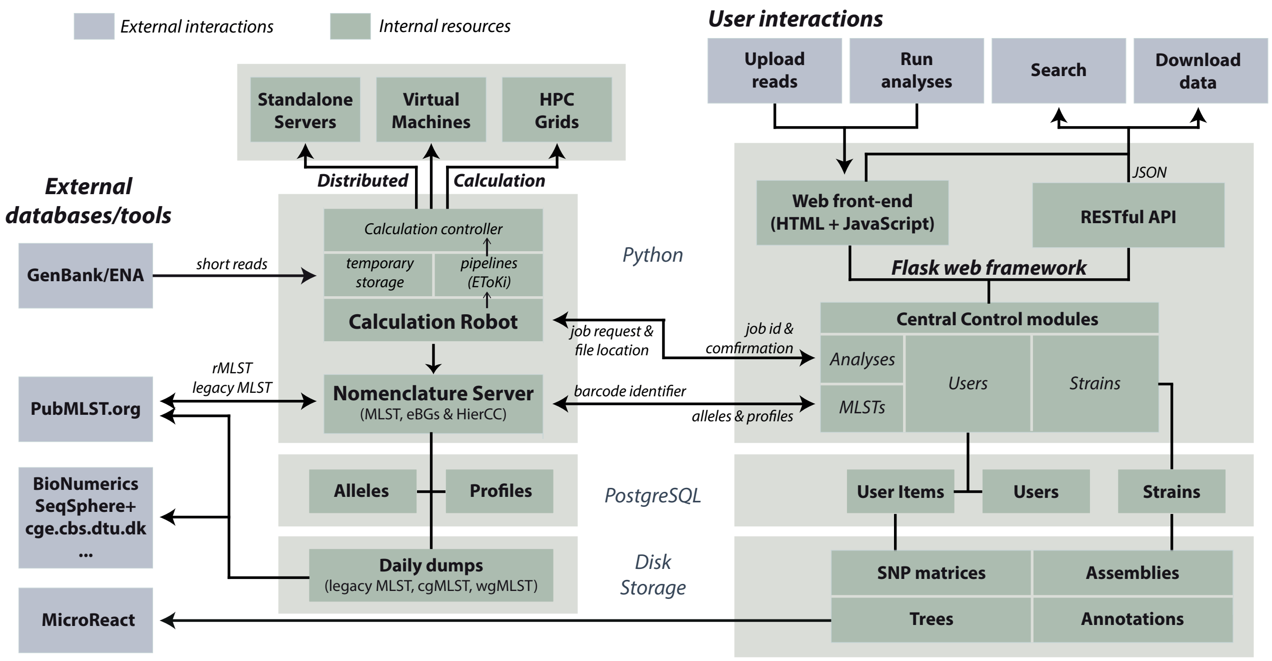

Supplemental Figure S1. Overview of workflow within EnteroBase. User interactions (upper right): EnteroBase supports user interactions via a web browser front end or a RESTful API (Application Programming Interface; details at https://enterobase.readthedocs.io/en/latest/api/about-the-api.html), both of which connect to a Python Flask web framework environment that handles all further interactions with structured data (PostgreSQL databases) and stored files in disk storage via Central Control modules. These Central Control modules are also responsible for interacting *via* JSON strings with the Calculation Robot and Nomenclature Server. The Calculation Robot is responsible for automatically uploading novel short read sequences from GenBank, and uses EToKi (Fig. S2) to assemble and annotate draft genomes, and perform other calculations. The Nomenclature Server (NServ) is responsible for defining and calling MLST alleles and Sequence Types (STs), as well as maintaining population structure designations and assignments. NServ also communicates with external databases; it exchanges data with PubMLST and provides daily dumps of legacy MLST, cgMLST and wgMLST data for external databases and commercial providers.

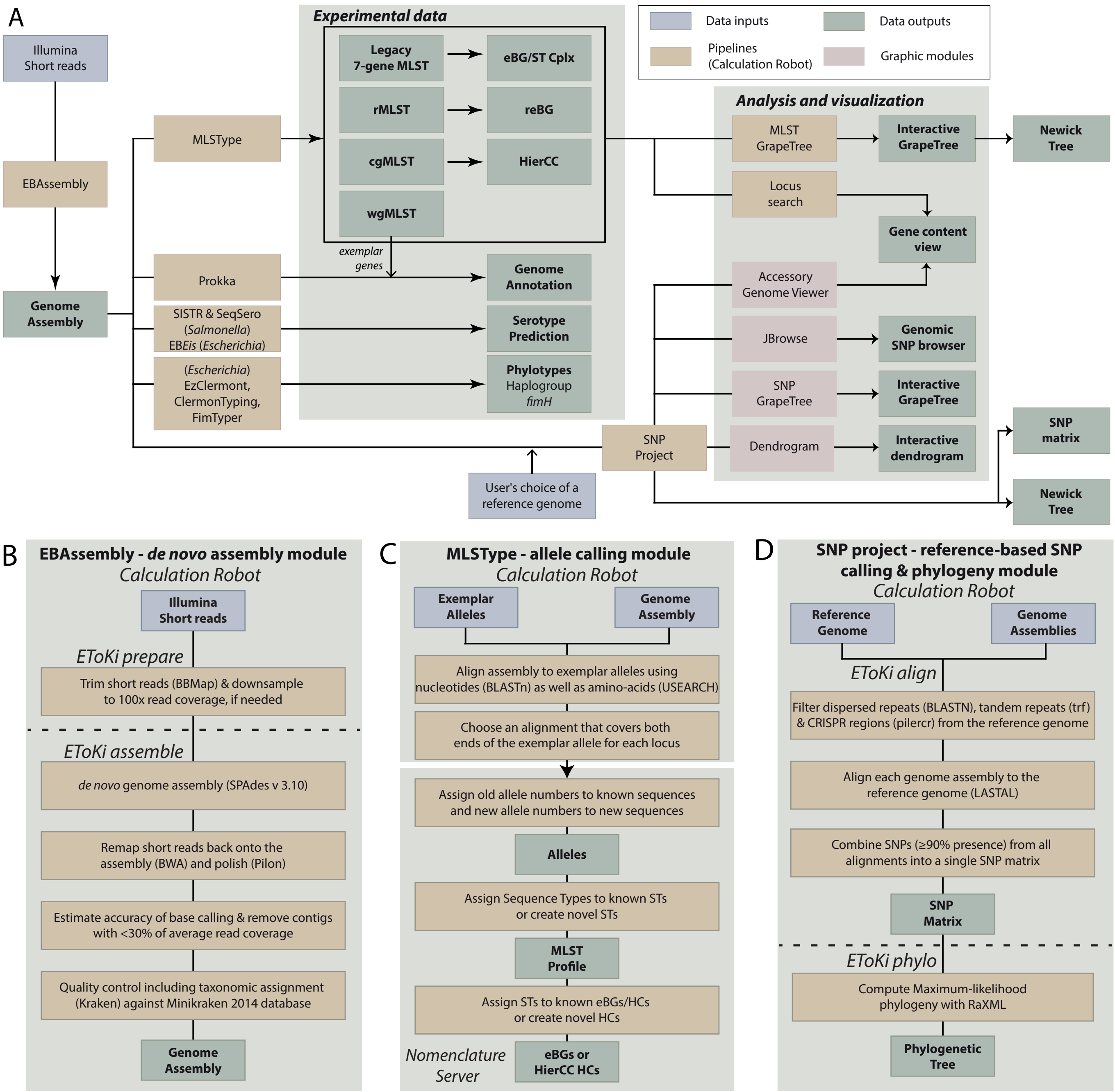

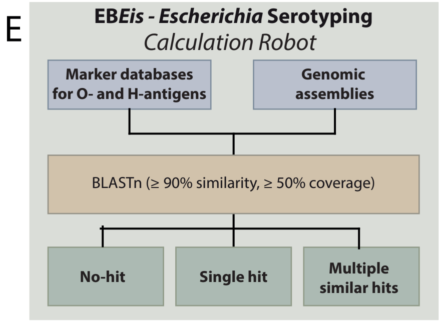

Supplemental Figure S2. EnteroBase algorithms and the EToKi toolkit. The backend pipelines within EnteroBase use multiple calculation and graphic modules, to produce a large variety of data and graphical outputs (https://enterobase.readthedocs.io/en/latest//about.html), including a variety of stand-alone programs. A) Algorithm flow. Left: Illumina short reads are assembled in genomes, which are then used for MLST typing at multiple levels of resolution, annotation, and the prediction of serotypes and phylotypes. Right: Tools for the analysis and visualization of genetic distances (MLST alleles or SNPs) and metadata for selected database entries. B-E) Four sets of modules within EToKi (EnteroBase ToolKit), a publicly available standalone package for external command line usage of EnteroBase pipelines that are potentially of general interest. B) EBAssembly performs trimming and automatic down-sampling from enormous datasets (EToKi prepare), followed by assemblies, remapping and polishing (EToKi assemble). EBAssembly estimates the accuracy of base calls and the most probable taxonomic sources of the sequences (Kraken against MiniKraken database (Wood and Salzberg 2014)). These summary statistics are presented in the EnteroBase Experimental Data field "Assembly stats". EBAssembly can also be used for extracting genomic assemblies from metagenomic data (see Supplemental Fig. S6). Standalone programs called by EBAssembly: BBMap in BBTools (Bushnell 2016), SPAdes (Bankevich et al. 2012), BWA (Li and Durbin 2010), Pilon (Walker et al. 2014). C) MLSType calls MLST alleles from genomic assemblies. EnteroBase maintains "Exemplar Alleles", consisting of one allele sequence for each gene in the wgMLST scheme and in legacy MLST. (rMLST and cgMLST are subsets of the wgMLST scheme.) The Exemplar Alleles are aligned to each genome assembly with BLASTn (Altschul et al. 1990) or the USEARCH module UBlastP (Edgar 2010) in order to map the ends of their corresponding loci and extract their sequences. These are assigned to existing allele numbers and STs for each of the MLST schemes unless they are novel, in which case new numbers are assigned. STs are assigned to known eBGs or HC clusters or used to define new clusters. In EnteroBase, these tasks are separately performed by the Calculation Robot and the Nomenclature Server. D) An EnteroBase SNP project first masks repetitive dispersed repeats (BLASTN) or tandem repeats (trf (Benson 1999)) or CRISPR regions (pilercr (Edgar 2007)) in the reference genome, and then aligns each genome assembly to that partially masked reference genome (LASTAL in Last (Kielbasa et al. 2011)) (EToKi align). The resulting SNP matrix can be downloaded by the user and/or used to calculate an ML phylogeny (RAxML V8 (Stamatakis 2014)) (EToKi phylo). Within EnteroBase, ML phylogenies can be calculated for up to 200 genomes and then displayed by GrapeTree or Dendrogram. SNP Project in EToKi is suitable for larger projects because the number of genomes is not limited except by computer hardware constraints. However, EToKi does not include graphical visualisation tools. E) EB*Eis* predicts *Escherichia* O serotypes based on the marker genes *wzx, wzy, wzt, and wzm* (Joensen et al. 2015; DebRoy et al. 2016) and H serotypes based on the flagellar *fliC* gene (Joensen et al. 2015). The standalone version of EToKi including the standalone programs cited above is available at GitHub (https://github.com/zheminzhou/EToKi) and in supplemental files.

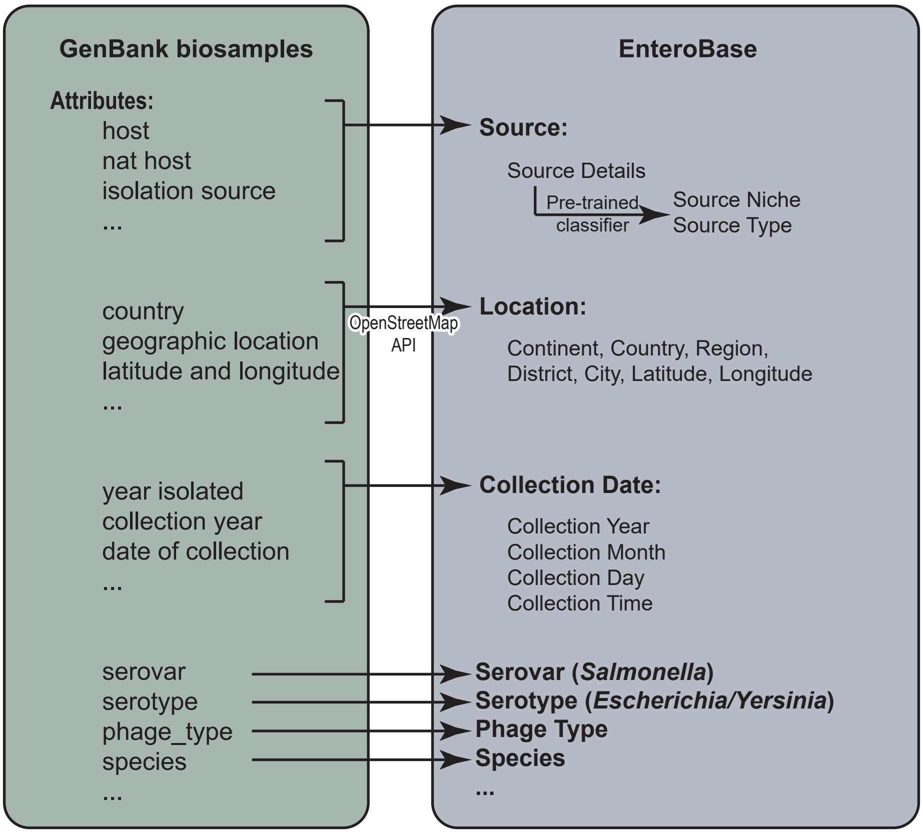

Supplemental Figure S3. Correspondences between metadata fields in GenBank and in EnteroBase. EnteroBase metadata fields (Supplemental Table S2) are somewhat different from metadata fields in GenBank. GenBank metadata in the categories Source, Location, Date and Others are therefore automatically converted by an automated metaparser into EnteroBase metadata categories (Supplemental Table S4) as part of the uploading process. EnteroBase stores the original text from GenBank Source in Source Details and classifies those contents into Source Niche and Source Type (Supplemental Table S2B), all of which are combined within the Source composite field (Supplemental Table S2A).The other categories of GenBank metadata correspond closely to EnteroBase metadata categories except that some of them are presented as composite fields within EnteroBase (Table S2A).

A

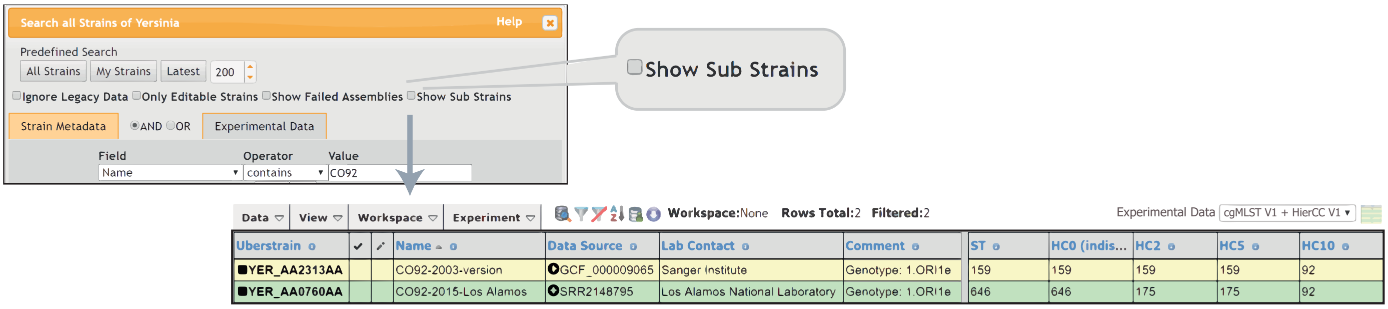

B

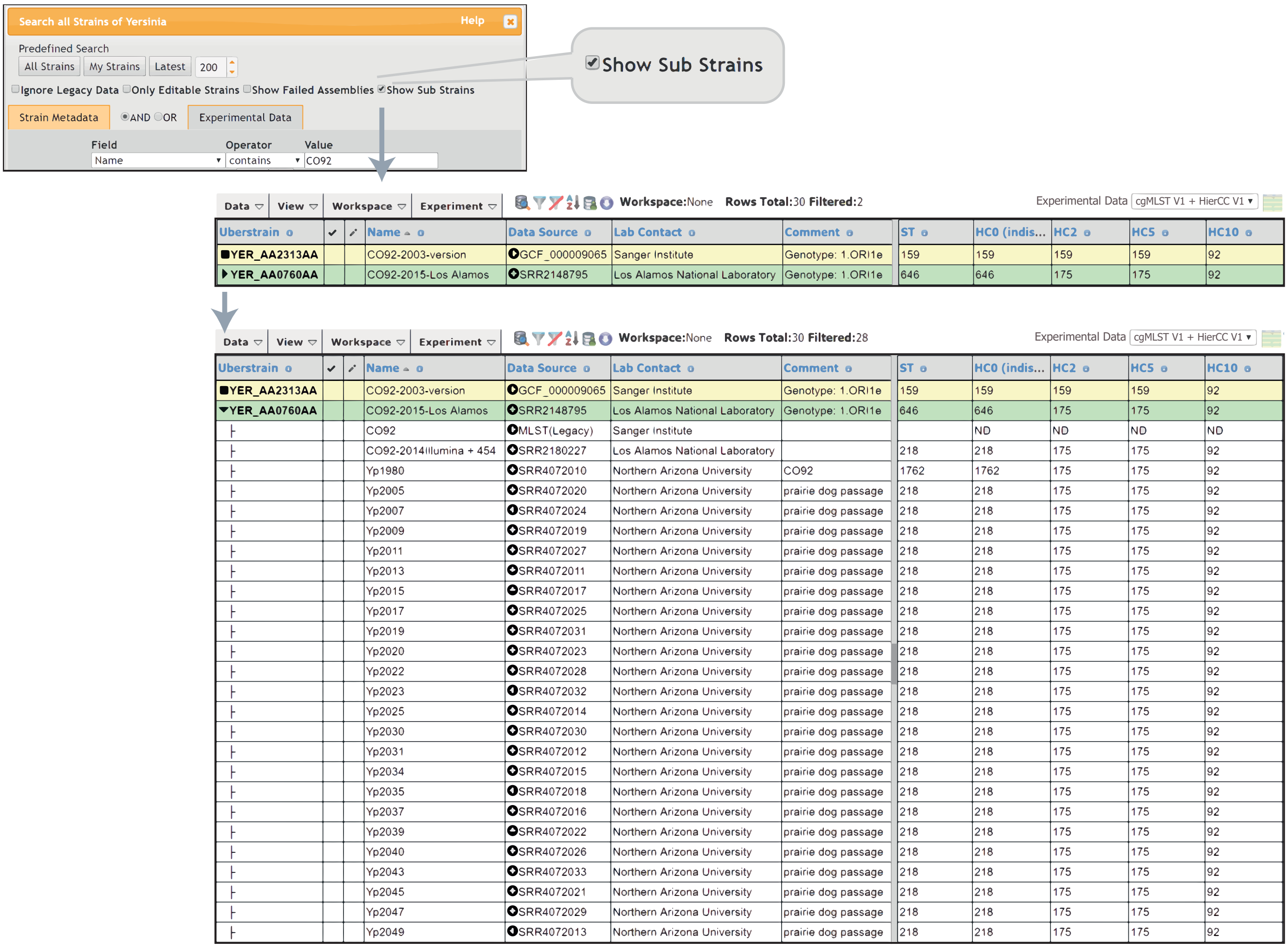

Supplemental Figure S4. Uberstrains and sub-strains. A) In its default mode, Show Sub Strains is unticked within the Search Dialog (top), and only Uberstrains are retrieved by the workspace (bottom), as indicated by a black square to the left of the Uberstrain barcode designation at the left. The example shows two distinct Uberstrains of *Y. pestis* CO92, one which was sequenced in 2001 and a second sequenced in 2015, in which 13 erroneous SNP calls have been corrected. B) When Show Sub Strains is checked in the search dialog, the browser shows a triangle at the far left of Uberstrains that contain one or more Sub-Strains. Clicking on that triangle opens a previously hidden tree-like hierarchy containing all its sub-strains. To open these hierarchies for all Uberstrains in the browser window, choose View\Show All Sub-strains in the top browser Menu. View\Close all Sub-strains reverts to showing only Uberstrains.

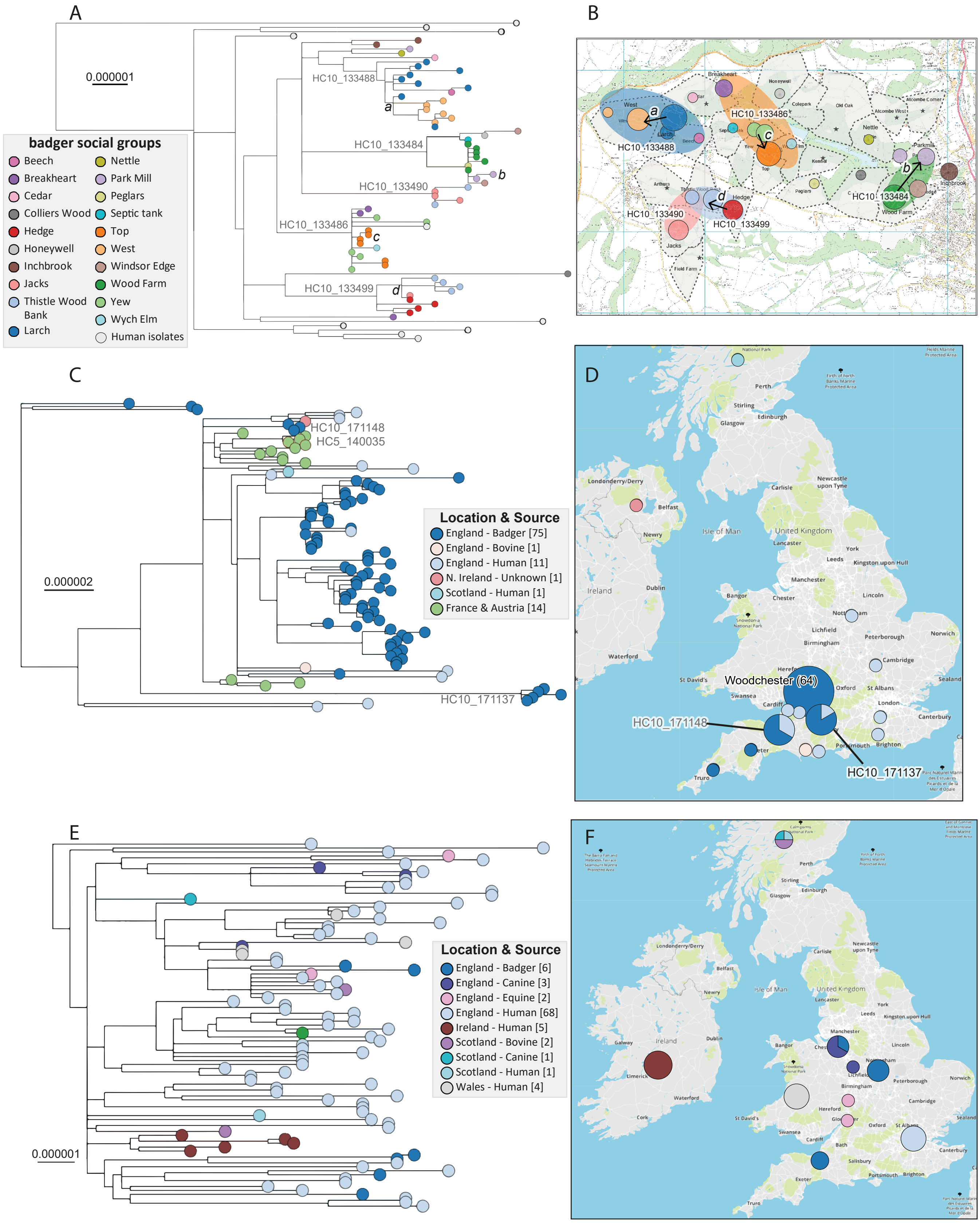

Supplemental Figure S5. Phylodynamics of isolates from badgers. GrapeTree was used to transfer individual subtrees plus their GPS coordinates and metadata to MicroReact (Argimon et al. 2016) (https://enterobase.readthedocs.io/en/latest/grapetree/grapetree-manual.html). (A, C, E) Phylogenetic trees drawn by MicroReact. (B, D, F) Maps of geographic locations within MicroReact, except that in part B, where the MicroReact tree was overlaid by the idealized spatial distributions of badger social groups and setts as elucidated by (McDonald et al. 2018). (A, B) Sixty four Agama isolates from badgers in Woodchester Park that were collected in 2006-2007 plus 10 related isolates from humans. Five HC10 clusters of genetically related genomes were isolated from neighbouring badger social groups (colored ovals in part B), of which four are inferred to have moved by local transmission chains a, b, c, d as indicated in part B (https://microreact.org/project/t7qlSSslh/3e634888). (C, D) 103 Agama isolates in HC100_2433, including 75 from badgers in Woodchester Park and elsewhere in England that were collected between 1998 and 2010 (https://microreact.org/project/9XUC7i-Fm/fed65ff5). (E, F) 92 Agama isolates in HC100_299 from the British Isles, including 6 from badgers that were collected between 2009 and 2016 (https://microreact.org/project/XaJm1cNjY/69748fe3).

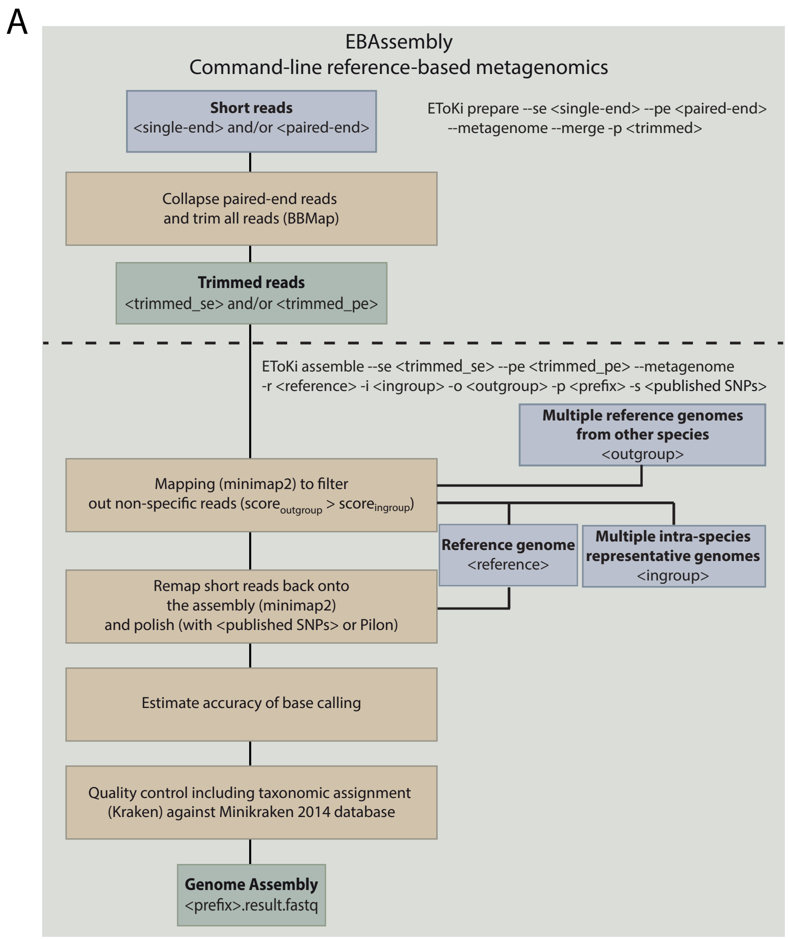

Supplemental Figure S6. Extracting aDNA assemblies from metagenomic sequences with the EBAssembly module of EToKi. EBAssembly includes functions for extracting genome-specific reads from metagenomic sequences which are only accessible in the stand-alone, command-line version of EToKi. The EToKi prepare module can collapse paired-end reads and trim both paired-end and single-end reads without down-sampling. As described in the documentation (https://github.com/zheminzhou/EToKi), the EToKi assemble module incorporates elements from SPARSE (Zhou et al. 2018; Achtman and Zhou 2019) to identify genome-specific short reads within metagenomic sequences after specifying a reference genome sequence, an in-group of related genomes and a related but distinct out-group of genomes . The module replaces nucleotides in the reference genome by their calculated SNVs after checking that they are supported by at least 3 metagenomic reads, and the supporting read frequencies occur with at least one-third of the average read depth. It also allows constraining SNP calls to (published) SNPS within a text file, and saves the modified sequence of the reference genome in a form which can be uploaded to EnteroBase by admins and curators.

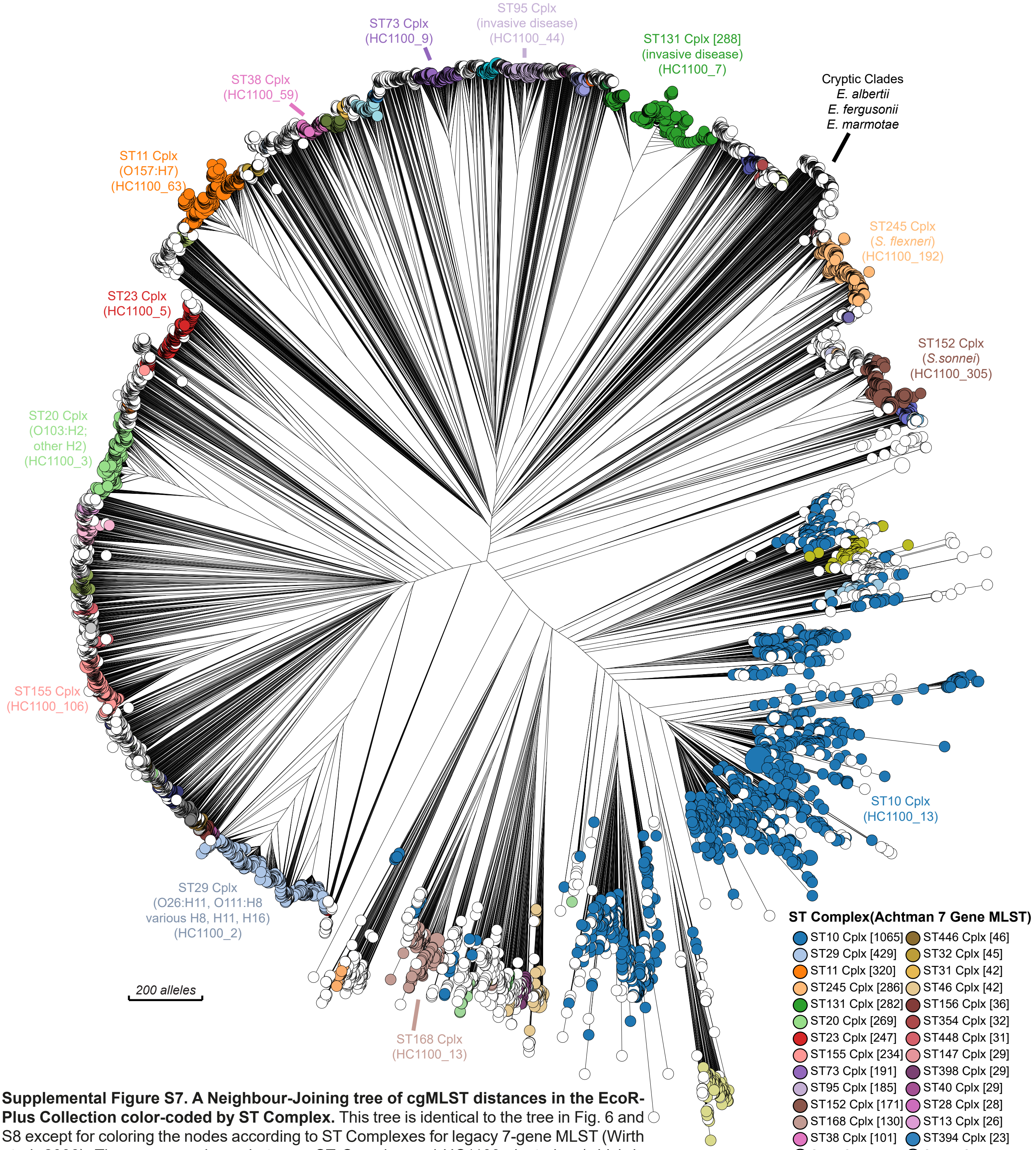

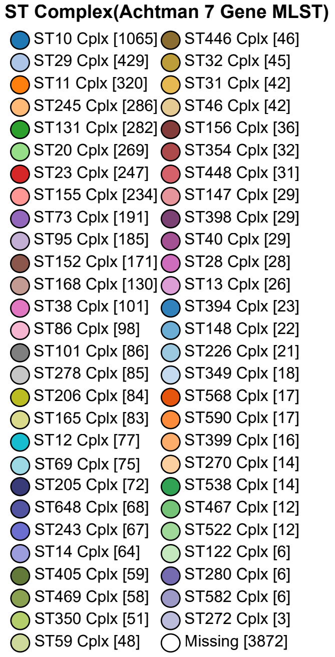

Supplemental Figure S7. A Neighbour-Joining tree of cgMLST distances in the EcoR-Plus Collection color-coded by ST Complex. This tree is identical to the tree in Fig. 6 and S8 except for coloring the nodes according to ST Complexes for legacy 7-gene MLST (Wirth et al. 2006). The correspondence between ST Complex and HC1100 clustering (which is based on much higher resolution cgMLST) is striking for the most common ST Complexes, with the exception of ST168 Complex (07:00) which is assigned to HC1100_13 by Hierarchical Clustering, and additional, rarer ST Complexes. The discrete nature of multiple ST Complexes according to cgMLST is noteworthy, and prominent examples of such discrete Complexes are indicated by their designations. Recent publications have provided interesting details on the ST131 Complex (usually erroneously referred to as ST131) (Johnson et al. 2016; Stoesser et al. 2016; Ben Zakour et al. 2016; Liu et al. 2018), the ST95 Complex (Achtman et al. 1983; Wirth et al. 2006; Gordon et al. 2017) and ST11 Complex/O157:H7 (Leopold et al. 2009; Dallman et al. 2015). Recent attention based on genomes in EnteroBase has been dedicated to ST1193 of ST14 Complex (Johnson et al. 2019). However, little attention has been directed at the other ST Complexes although they are a very common cause of disease in humans and animals according to the frequencies of their genomes in EnteroBase.

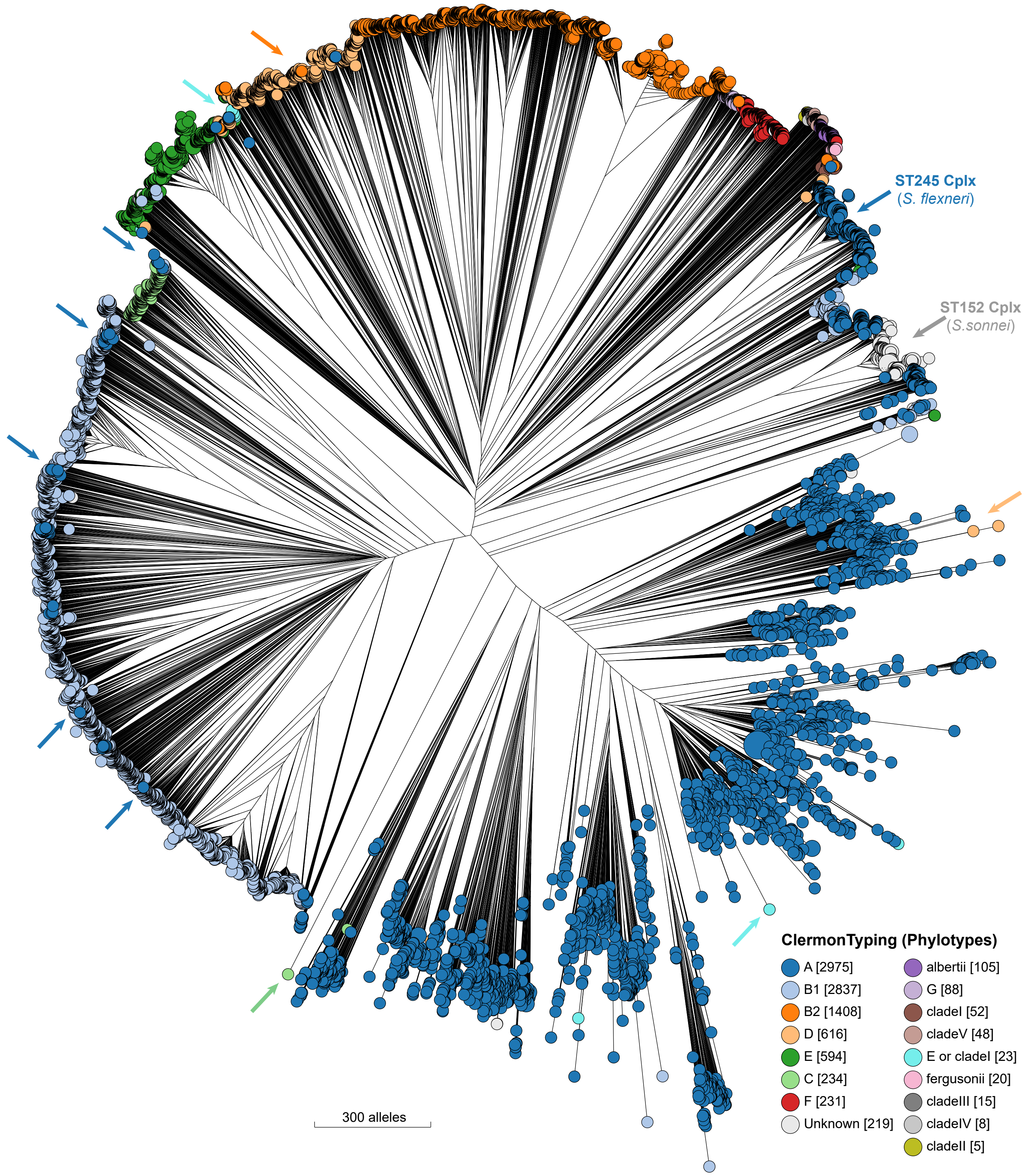

Supplemental Figure S8. A Neighbour-Joining tree of cgMLST distances in the EcoRPlus Collection color-coded by Clermont types. This tree is identical to the tree in Fig. 6 and Fig. S7 except that the nodes show the Clermont Types predicted by the program ClermonTyping (Beghain et al. 2018), which has been implemented within EnteroBase. Large parts of the tree are relatively homogeneous, indicating that Clermont Typing often correlates well with HC2000 clustering. However, arrows indicate multiple nodes which differ in Clermont Type from their close neighbors, illustrating that the presence/absence of genes from the accessory genome which is used for the Clermont scheme does not correlate completely with the phylogenetic relationships revealed by cgMLST. As a result, nodes assigned to Clermont Types A and B2 are found at multiple positions within the tree, far from most other strains of those Clermont types. In addition, two groups of Shigella are inaccurately labelled by Clermont Types. ST245 Complex largely corresponds to *Shigella flexneri* (Wirth et al. 2006), but is inappropriately assigned to Clermont Type A. Similarly, ST152 Complex largely corresponds to Shigella sonnei but is not recognized by Clermont Typing.

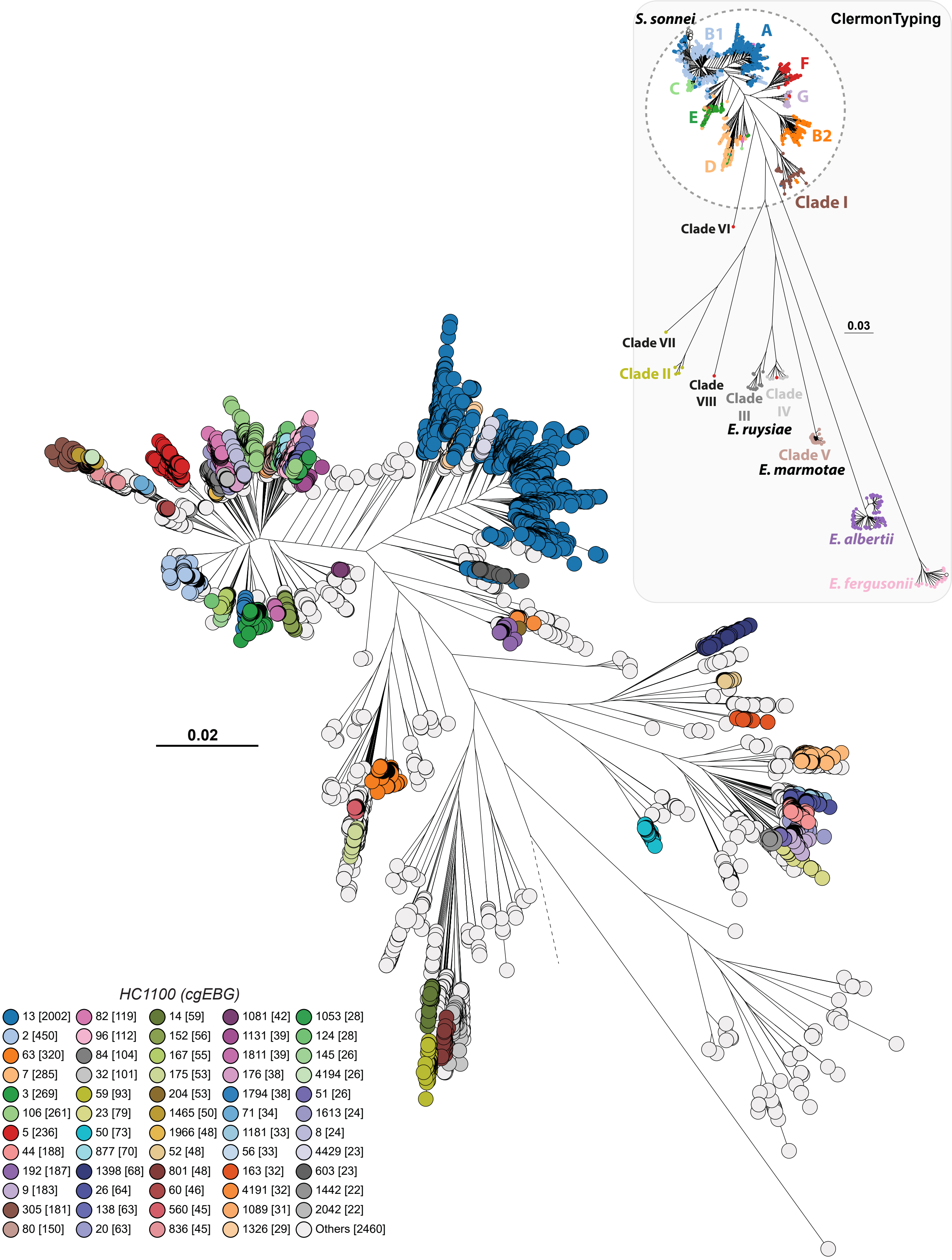

Supplemental Figure S9. A Maximum-Likelihood (ML) tree of the EcoRPlus Collection. 1,230,995 core SNPs were extracted from 9,479 core genomes after concatenating the sequences (2.33 Mbps) of their 2,513 core gene alignments with MAFFT (Katoh and Standley 2013). A maximum likelihood tree was calculated using FASTTREE 2 (Price et al. 2010). **Inset**) The ML tree of all genomes color-coded by ClermonTyping, including the Cryptic Clades I-VI, two novel cryptic clades (VII-VIII) and the *Escherichia* species *albertii, fergusonii*, and *marmotae*. Note that the three genomes of *E. marmotae* are on a deep branch within Clade V, in agreement with independent recent observations (van der Putten et al. 2019), which also assigned Clades III plus IV to the novel species *Escherichia ruysiae*. The white circle encloses genetically-related populations within *E. coli*, including Clade I, whereas the other Clades and species are on external branches within the gray rectangle. These topological relationships are similar to those described on smaller datasets (Luo et al. 2011; van der Putten et al. 2019). **Main figure**) Closeup of genomes on branches within the inner circle of the inset, color-coded by HC1100 HierCC cluster. This ML clustering of individual genomes is concordant with the clustering according to the neighbour-joining algorithm in Fig. 6, but provides much more accurate branch lengths. However, this tree also took several weeks to complete whereas Fig. 6 was complete in less than an hour.

Supplemental Table S1. Details of quality controls settings that are used by Enterobase for draft assemblies.

Genus	min size (KBps)	max size (KBps)	min N50 (KBps)	max number of contigs	max low quality sites	min taxonomic purity
Salmonella	4,000	5,800	20	600	5%	70%
Escherichia/ Shigella	3,700	6,400	15	800	5%	70%
Yersinia	3,700	5,500	15	600	5%	65%
Clostridioides	3,600	4,800	20	600	5%	65%
Moraxella	1,800	2,600	20	600	5%	65%
Helicobacter	1,300	3,000	10	600	5%	65%
Vibrio	3,000	5,900	20	700	5%	65%

Supplemental Table S2A. Metadata fields used in all EnteroBase databases.

Field	Description	Special features and editing format
Uberstrain	Primary entry	Place holder for multiple almost identical entries
Name	Strain designation	Not necessarily unique
Comment	Free text	Unrestricted text
Data Source[1]	Properties of short reads	Includes Accession number. Special editing dialog box
Source-> Source Niche[2]	Ecological niche	Dropdown list (Human, Aquatic, Food, etc.)
Source-> Source Type[2]	Taxonomy of source	Dropdown list (Human, Avian, Camelid, etc.)
Source-> Source Details[2]	Free Text	Additional details
Collection Date-> Year[3]	Year of sample	Text with calendar date sub-dialog
Collection Date-> Month[3]	Month of sample	Text with calendar date sub-dialog
Collection Date-> Day[3]	Day of sample	Text with calendar date sub-dialog
Collection Date-> Time[3]	Time of sample	Text
Location-> Continent[4]	Geographical data	Text with browser suggestions. Google map.
Location-> Country[4]	Geographical data	Text with browser suggestions. Google map.
Location-> Region[4]	Geographical data	Text with browser suggestions. Google map.
Location-> District[4]	Geographical data	Text with browser suggestions. Google map.
Location-> City[4]	Geographical data	Text with browser suggestions. Google map.
Location-> Post Code[4]	Geographical data	Text. Google map
Location-> Latitude[4]	Geographical data	Text. Google map.
Location-> Longitude[4]	Geographical data	Text. Google map.
Lab Contact	Free Text	Institution for contact
Species	Species or sub-species	Dropdown list
Project-> Bio Project ID[5]	NCBI BioProject Accession	Non-editable URL
Project-> Project ID[5]	NCBI SRA Study	Non-editable URL
Sample-> Sample ID[6]	NCBI BioSample Accession	Non-editable URL
Sample-> Secondary Sample ID[6]	NCBI SRA Secondary Sample	Non-editable URL
Date Entered	Initial publication date	Non-editable information
Release Date	Future release date (<12 m)	User-specified delay for public downloads
Barcode	Internal EnteroBase ID	Unique identifier for each database entry

NOTES:

[1]Data source has the format (Accession No.;Sequencing Platform;Sequencing Library;Insert Size;Experiment;Status) when downloaded

[2]Composite of multiple sub-fields. To select default field right click on Header "Source".

[3] Composite of multiple sub-fields. Default field selectable through right click on Header "Collection Date. To edit year, month and day, click on Calendar icon.

[4]Composite of geographical data supported by a Google Maps depiction. Same format as MicroReact. To select default field right click on Header "Location".

[5]Composite of Project information as stored at GenBank/NCBI. To select default field right click on Header "Project".

[6]Composite of Sample information as stored at GenBank/NCBI. To select default field right click on Header "Sample".

Supplemental Table S2B. Database-specific fields.

Field	Description	Special features and editing format
Escherichia/Shigella	Legacy fields from legacy MLST	
Serological Group	Common descriptor e.g. K1	Dropdown list of common names
Serotype	Antigenic formula	Text box
EcoR Cluster	Haplogroup designation	Dropdown list
Path/Nonpath	Pathogen or Non pathogen	Dropdown list
Simple Patho	Acronyms for pathovars	Dropdown list
Simple Disease	Clinical disease category	Text box
Disease	Clinical disease category	Dropdown list
Salmonella		
Disease	Clinical disease category	Text box
Antigenic Formulas	O:H1:H2 formula	Text box
Phage Type	Phage typing designation	Text box
Serovar	Kaufmann-White Serovar name	Text box with browser suggestions
Subspecies	I, II, …VII, *S. bongori*, novel subsp. A-C	Dropdown list
Clostridioides		
PCR Ribotype	Classical ribotype designation (3-digits)	Text box
Helicobacter		
Alias	Alternative strain name	Text box
Antimicrobial Resistance	List of resistances	Text box
Citations	PubMed IDs	Text box
Contact	Name and/or Email address of contact person	Text box
Source-> Host Age	Age of carrier	Multiline dialog. Text box.
Source-> Host Ethnicity	Ethnicity of carrier	Multiline dialog. Text box.
Source-> Host Sex	Sex of carrier	Dropdown list
Uploader	Lab source of data	Text box
Yersinia	Legacy fields from legacy MLST	
Disease	Clinical disease category	Text box
Alternative Name	Alternative strain name	Text box
Biogroup	Sub-species based on phenotype, e.g. Orientalis	Text box
Contact Name	Laboratory who uploaded reads	Text box
O Serotype	Serological formula	Text box
Moraxella	Legacy fields from legacy MLST	
Path/Nonpath	Pathogen or Non pathogen	Dropdown list

Supplemental Table 3a. Summary of *Salmonella enterica* serovar Agama HC2000_299 isolates

Categories	England	Scotland	Wales	Northern Ireland	Ireland	France	Austria	Germany	Netherlands	Nigeria	United States	Unknown	Summary
**Human**	127 (2012-2018)	7 (1998-2018)	4 (2014-2015)	-	20 (2006-2017)	33 (2000-2018)	6 (2000-2018)	-	1 (2006)	6 (2012-2013)	-	-	204 (1998-2018)
**European badger**	81 (1998-2016)	-	-	-	-	-	-	-	-	-	-	-	81 (1998-2016)
**Bovine**	1 (2009)	2 (2003,2018)	-	-	1 (2010)	-	-	1 (2018)	-	1 (2014)	-	-	6 (2003-2018)
**Equine**	3 (2015)	-	-	-	-	-	-	-	-	-	-	-	3 (2015)
**Canine**	4 (2014-2015)	1 (2003)	-	-	-	-	-	-	-	-	-	-	5 (2003-2015)
**Food/Animal Feed**	-	-	-	-	1 (2007)	-	2 (2014-2018)	1 (1999)	-	1 (2016)	-	-	5 (1999-2018)
**Poultry**	-	-	-	-	-	-	-	-	-	5 (2011-2016)	-	-	5 (2011-2016)
**Swine**	1 (2015)	-	-	-	-	-	-	1 (2007)	-	2 (2011-2014)	-	-	4 (2007-2015)
**Reptile**	-	-	-	-	-	-	1 (2000)	2 (2006, 2008)	-	1 (1959)	-	-	4 (1959-2008)
**Mussels**	-	-	-	-	1 (2007)	-	-	-	-	-	-	-	1 (2007)
**Environment / Unknown**	1 (2018)	1	-	1 (2006)	-	-	1 (2000)	1 (2015)	-	-	2	3	10 (2000-2018)
**Summary**	218 (1998-2018)	11 (1998-2018)	4 (2014-2015)	1 (2006)	23 (2006-2017)	33 (2000-2018)	10 (2000-2018)	6 (1999-2018)	1 (2006)	16 (1959-2016)	2	3	328 (1959-2018)

Supplemental Table 3b. Summary of *Salmonella enterica* serovar Agama HC400_299 isolates

Categories	England	Scotland	Wales	Northern Ireland	Ireland	France	Austria	Germany	Summary
**Human**	83 (2012-2018)	2 (1999,2003)	4 (2014-2015)	-	15 (2008-2017)	12 (2010-2018)	1 (2018)	-	117 (1999-2018)
**European badger**	81 (1998-2016)	-	-	-	-	-	-	-	81 (1998-2016)
**Bovine**	1 (2009)	2 (2003,2018)	-	-	1 (2010)	-	-	-	4 (2003-2018)
**Equine**	2 (2015)	-	-	-	-	-	-	-	2 (2015)
**Canine**	3 (2014-2015)	1 (2003)	-	-	-	-	-	-	4 (2003-2015)
**Food/Animal Feed**	-	-	-	-	-	-	2 (2018)	-	2 (2018)
**Mussels**	-	-	-	-	1 (2007)	-	-	-	1 (2007)
**Environment / Unknown**	-	-	-	1 (2006)	-	-	-	1 (2015)	2 (2006,2015)
**Summary**	170 (1998-2018)	5 (1999-2018)	4 (2014-2015)	1 (2006)	17 (2007-2017)	12 (2010-2018)	3 (2018)	1 (2015)	213 (1998-2018)

Supplemental Table S4. EnteroBase metaparser classification scheme for Source Niche and Source Type based on Source Details

Source Niche	Source Type	Examples of Source Details
Aquatic	Fish; Marine Mammal; Shellfish	Tuna, lobster
Companion Animal	Canine; Feline	Cat, dog
Environment	Air; Plant; Soil/Dust; Water	River, tree, soil
Feed	Animal Feed; Meat	Dog treat, fishmeal
Food	Composite Food; Dairy; Fish; Meat; Shellfish	Milk, salami, ready-to-eat food
Human	Human	Patient, biopsy
Laboratory	Laboratory	Reference strain, serial passage
Livestock	Bovine; Camelid; Equine; Ovine; Swine	Horse, calf
Poultry	Avian	Turkey, chicken
Wild Animal	Amphibian; Avian; Bat; Bovine; Camelid; Canine; Deer; Equine; Feline; Invertebrates; Marsupial; Other Mammal; Ovine; Primate; Reptile; Rodent; Swine	Flamingo, frog, python, Spider
ND	ND	

Supplemental Table S5. Four HC5 clusters of *E. coli* that were isolated from multiple dates, locations and hosts, including Seagulls.

HC5 Cluster	ST Complex	EnteroBase Barcode	Strain name	Isolation Date	Source	Location
116782	ST10 Cplx	ESC_OA6857AA	1195_C3G	2016-8	Seagull	USA: Alaska
		ESC_OA6822AA	1238_C3G	2016-8	Seagull	USA: Alaska
		ESC_OA5258AA	NYVetLIRN-185	2019-9	Chicken	USA: New York
1237	ST131 Cplx	ESC_OA6294AA	108L-Seagull		Seagull	
		ESC_OA6296AA	150L-crow		Crow	
		ESC_OA6299AA	229L-crow		Crow	
		ESC_OA5132AA	75845	2007-10	Human	Canada: Quebec
		ESC_OA7568AA	96859	2011-2	Human	Canada: Quebec
		ESC_CA9494AA	VRES0235			
74116	ST155 Cplx	ESC_OA6777AA	411_Uriselect	2016-6	Seagull	USA: Alaska
		ESC_OA6833AA	409_Uriselect	2016-6	Seagull	USA: Alaska
		ESC_KA1867AA	FSIS11813959	2018	Swine	USA: Michigan
71128	ST38 Cplx	ESC_JA8566AA	312ESBA	2017	Seagull	Australia: Tasmania
		ESC_JA8605AA	298ESBA	2017	Seagull	Australia: Tasmania
		ESC_OA1888AA	AUSMDU00008632	2017	Human	Australia
		ESC_OA1762AA	AUSMDU00008001	2017	Human	Australia
		ESC_OA1711AA	AUSMDU00008259	2017	Human	Australia
		ESC_OA1720AA	AUSMDU00008492	2017	Human	Australia
